# Actigraphy-derived multidimensional sleep health among breast cancer survivors and controls: Pink SWAN

**DOI:** 10.1007/s11764-024-01715-y

**Published:** 2024-11-30

**Authors:** Sarah N. Price, Sybil L. Crawford, Leslie M. Swanson, Michelle M. Hood, Nancy E. Avis

**Affiliations:** 1 Department of Social Sciences and Health Policy, Wake Forest University School of Medicine, 525 Vine Street, Ste. 410, Winston-Salem, NC 27101 USA; 2 Tan Chingfen Graduate School of Nursing, UMass Chan Medical School, Worcester, MA USA; 3 Department of Psychiatry, University of Michigan School of Medicine, Ann Arbor, MI USA; 4 Department of Epidemiology, School of Public Health, University of Michigan, Ann Arbor, MI USA

**Keywords:** Sleep, Breast cancer, Cancer survivors

## Abstract

**Purpose:**

To compare breast cancer survivors (BCS) to women without breast cancer (controls) on sleep health risk factors and actigraphy-derived dimensions of sleep (duration, maintenance, timing, and regularity) and examine whether the effect of breast cancer on sleep differs by time since diagnosis.

**Methods:**

Analyses included data from 68 BCS and 1042 controls who participated in actigraphy and Pink SWAN sub-studies within the Study of Women’s Health Across the Nation. BCS and control characteristics were compared using chi-square, Fisher’s exact, and Wilcoxon rank sum tests. Sleep measures were regressed onto breast cancer status using binomial logistic and linear regression. The interaction between BCS status and years since diagnosis (< 5; ≥ 5) was tested in these models before and after covariate adjustment.

**Results:**

There were no overall sleep differences between BCS and controls; both groups experienced poor sleep health on average across multiple dimensions. Physical inactivity, sleep apnea, and vasomotor and depressive symptoms were associated with worse sleep in both groups. Total sleep time was lower among BCS than controls within 5 years of diagnosis (6.13 vs. 6.57 h; *p* = .03) but did not differ at > 5 years post-diagnosis (6.59 vs. 6.45 h; *p* = .32). BCS reported greater use of exogenous hormones (*p* < .0001) and were twice as likely to have initiated anxiolytic use post-diagnosis (*p* = .03).

**Conclusions:**

BCS within 5 years of diagnosis experienced shorter sleep duration than controls but did not differ on other sleep parameters. Both groups experienced poor sleep health.

**Implications for Cancer Survivors:**

BCS and similarly-aged women experience poor sleep health requiring assessment and treatment.

## Background

Sleep disturbances are among the most common and bothersome issues reported by breast cancer survivors (BCS) during and after treatment [1]. The estimated prevalence of sleep disturbances (inclusive of insomnia symptoms, poor sleep quality, and sleep–wake activity rhythm disturbance [2]) among BCS is 40% but ranges widely according to the type of sleep parameter assessed and the assessment method used [3]. As many as 85% of recently diagnosed BCS report insomnia symptoms [4] and smaller yet sizeable proportion of BCS (~ 15–36%) experience symptoms that rise to the level of insomnia syndrome [5–7]. Longitudinal and cross-sectional studies of BCS have found that anti-cancer treatments disrupt sleep–wake activity rhythms [8–10] and that the period following diagnosis is associated with high rates of sleep disturbance which diminish but do not completely resolve 12–18 months following treatment initiation [4–7]. A significant proportion (approximately 40%) of disease-free BCS 1–10 years post-treatment report poor quality sleep [11] and a need for assistance in addressing sleep problems [12], thus providing indirect evidence that sleep issues experienced during treatment may become chronic for a substantial percentage of BCS. Multiple mechanisms unique to breast cancer (e.g., direct tumor effects, effects of toxic agents, interferences of other cancer-related symptoms, and side effects) appear to disturb sleep in this population [7, 13, 14]. However, the degree to which breast cancer diagnosis and treatment may disturb sleep above and beyond other factors experienced by women in mid-life is unclear. Poor sleep health is commonplace in the general population of women during the perimenopausal period [15], and approximately one quarter of BCS has been shown to exhibit insomnia symptoms prior to diagnosis [5].

Limited, and contradictory, studies exist comparing dimensions of sleep health among BCS and similarly-aged women without cancer. Some studies have found higher rates of poor *subjective* sleep health (worse global sleep quality, shorter duration, longer latency, worse maintenance, higher prevalence of sleep disorders, and greater insomnia severity) among BCS compared to non-cancer controls [9, 10, 16–19], while others have not (including longitudinal analyses of subjective sleep maintenance from the Study of Women’s Health Across the Nation [SWAN], and the Women’s Health Initiative) [20, 21]. It is important to note that these studies all used different measures of self-reported sleep health (Pittsburgh Sleep Quality Index, Insomnia Severity Index, and single item measures), and most focused on BCS in the period shortly following diagnosis.

To our knowledge, no studies have compared BCS and controls on sleep timing or sleep regularity using actigraphy, despite increasing recognition that regularity and timing represent key facets of sleep health [22–25]. Further, the few studies that have compared BCS and controls on actigraphy-derived measurements of sleep health have small (all but one < 40 BCS and all < 60 controls), relatively racially homogeneous samples (all but one > 85% white) [9, 10, 26, 27]. Extant studies comparing BCS and controls on actigraphy-derived nocturnal sleep measures found conflicting results. Some studies found that BCS and women without cancer did not differ on actigraphy-derived sleep duration [9, 10, 26], while another found a transient reduction in sleep duration following chemotherapy initiation [27]. One study found no differences in sleep initiation or sleep maintenance between BCS who were at least 6 months post-treatment and controls [10], while another found that BCS within 1 year of active treatment experienced greater impairments in both of these sleep parameters compared to controls [26]. It is also unclear the degree to which decrements in sleep health (if there are any compared to controls) persist among BCS who are more than 1 year post-diagnosis, since longitudinal studies indicate a resolution of actigraphy-assessed sleep disturbances during or shortly after treatment [9, 27], while one of the aforementioned cross-sectional studies of BCS mostly post-treatment and on average more than 1 year post-diagnosis suggests that actigraphy may still detect some differences [26]. Given limited and contradictory published research, a more comprehensive investigation of differences in actigraphy-derived dimensions of sleep health between BCS and controls (inclusive of multiple important sleep dimensions like timing and regularity and controlling for relevant covariates) may yield insights to inform behavioral sleep interventions for this population by identifying aspects of sleep health that may require greater emphasis for the growing population of long-term BCS.

As a sleep assessment method, actigraphy offers unique information to enhance our understanding of potential differences between BCS and controls. Actigraphy can offer complementary and potentially more accurate information than self-reported measures regarding aspects of sleep such as regularity (variability night-to-night) [28] and maintenance (e.g., number and duration of awakenings) [29]. Coupled with sleep diaries, actigraphy is concordant with the “gold standard” objective sleep measure (polysomnography; PSG) and offers advantages over PSG as a low-cost naturalistic sleep observation method [28]. In the present study, we focused on the specific multidimensional sleep health domains from Buysse’s sleep health framework that can be derived from actigraphy [22, 30]: duration, maintenance (i.e., continuity and efficiency), regularity, and timing, offering an approach focused on those domains likely to be most impactful to health [31].

The present study used data from SWAN, a large longitudinal multisite study with rigorous survey and actigraphy data collection methods [32]. Because the majority of the BCS in SWAN were several years post-diagnosis at the time of actigraphy data collection, these comparisons provide insight into the degree to which diagnosis and treatment may have long-lasting impacts on key facets of sleep health in post-treatment survivors. Objectives of the present study were to (1) determine whether BCS differed from women without a history of breast cancer from the same cohort on multiple actigraphy-estimated dimensions of sleep health (duration, maintenance, regularity, and timing) in unadjusted and adjusted models, accounting for potential risk factors (e.g., age, sleep medication use, and vasomotor symptoms) and (2) test whether the effect of breast cancer status on sleep differed by time since diagnosis. We also conducted post hoc analyses examining associations between covariates and actigraphy-estimated dimensions of sleep in the adjusted models conducted for objective 1.

## Methods

### Sample and study overview

SWAN is a multiracial/ethnic cohort study originally designed to characterize the biological and psychosocial changes occurring during the menopausal transition [32]. From 1995–1997, seven clinical sites recruited non-Hispanic white women and women from one of four other racial/ethnic groups (Black, Japanese, Hispanic, or Chinese) and 3302 enrolled. The protocol was approved by Institutional Review Boards at each site. All participants provided written informed consent.

SWAN eligibility (assessed at enrollment) included age 42–52 years; an intact uterus and at least one ovary; not pregnant, lactating, using oral contraceptives, or hormone therapy; and having a menstrual cycle in the 3 months before screening [32]. Participants were assessed in-person at baseline and approximately annually through follow-up visit 15 in 2015–2016, using a standardized protocol that included interviews, clinical assessments, questionnaires, anthropometric measurements, and biological sample collection to assess medical, reproductive, and menstrual history; lifestyle and psychosocial factors; and physical and psychological symptoms. Instruments were translated into Spanish, Japanese, and Cantonese.

#### SWAN actigraphy sub-study

An actigraphy sub-study was conducted at SWAN visit 15 to assess sleep and physical activity (details on data collection are described under “Actigraphy and daily diaries”). Of the 2,091 women still in SWAN (66.3% of initially enrolled sample), a subset (1,333 women; 63.7%) participated in the actigraphy sub-study. Not all SWAN participants were invited to participate due to study and time constraints; each site was tasked with recruiting 141–225 women. Black, Chinese, Japanese, and Hispanic women were preferentially recruited to ensure diversity in the actigraphy sub-study. Exclusion criteria for the sub-study were use of a wheelchair, blindness, and plans to travel across time zones during protocol period. Participants in the sub-study did not differ from other SWAN participants at visit 15 on self-reported sleep characteristics [33].

#### Pink SWAN

Pink SWAN is another SWAN sub-study, focused on women who developed incident breast cancer (breast cancer survivors; BCS) during the years of SWAN follow-up. Pink SWAN was formed at visit 12 to allow for long-term follow-up of BCS and comparison to controls in a generalizable sample. Pink SWAN *cases* are women who developed *incident breast cancer* following SWAN enrollment and had no other prior cancer. Beginning at visit 12, we requested medical records from women reporting incident breast cancer; records were reviewed for cancer diagnosis and treatment, and we identified 151 incident cases. Of 110 cases for whom medical records were received, 104 of those breast cancers were confirmed, a 94.5% agreement between self-report and medical record. The remainder of self-reported cases were adjudicated by a medical oncologist. Date of diagnosis in BCS was the time “anchor” (i.e., time since diagnosis = 0) for analyses. To assign a corresponding date in controls, a date we refer to below as a “pseudo date of diagnosis,” we randomly assigned an index visit as the first post-diagnosis visit such that the distribution of first post-diagnosis visits was comparable in cases and controls, consistent with previous work [20, 34–43]. Pink SWAN *controls* (*N* = 2,161) included all participants in SWAN who had no breast cancer at baseline or during follow-up through visit 15 and who did not have a diagnosis of cancer other than breast on or prior to their randomly assigned “index visit.”

#### Analytic sample

The analytic sample for the current study consisted of women in Pink SWAN *and* the actigraphy sub-study, providing a sample of 68 BCS and 1,042 controls. Of 2,161 Pink SWAN controls, 1,119 were excluded because they did not participate in visit 15 actigraphy, and 108 were excluded for insufficient participation. Of 151 Pink SWAN BCS, 83 were excluded (79 did not participate in visit 15 actigraphy; 4 had insufficient participation). Data for the present cross-sectional analyses were primarily collected from visit 15.

### Procedures

#### Actigraphy and daily diaries

The wrist-worn Actiwatch-2 (AW-2, Philips Respironics, Bend, OR) was used to characterize sleep–wake patterns. Participants wore actigraphs on their non-dominant wrist for approximately 7 consecutive days and completed daily diaries in the morning and evening each day over the same period. Diaries queried participants about the times daily activities (e.g., sleep/work/exercise) started and ended, any sleep medications or naps they took, and any interruptions to their sleep. Participants were instructed to wear the Actiwatch-2 continuously and were provided guidance on how to complete the diaries, how to press the actigraph event marker to indicate going to bed with the intention of going to sleep and getting out of bed for the day.

The AW-2 accelerometer was set at 0.05 g for 3–11 Hz. The analog signal was digitized by the digital integration method. The wake threshold was set at 40 counts per minute, and data were sampled in 1-min epochs. Data were processed, evaluated for quality, and scored at the University of Pittsburgh study site with the sleep diary “in-hand” in conjunction with the standard sleep detection algorithm in Actiware 5.0.9 (Philips Respironics), using procedures consistent with Society of Behavioral Sleep Medicine guidelines [44]. Start and stop times were initially detected by the Actiware algorithm and hand-edited when needed to adjust misidentified sleep–wake times, with event markers used as a first choice over sleep diaries for marking the rest interval where possible. A minimum of 4 nights of actigraphy recording was required for data to be considered valid. Most of the women who participated in the actigraphy sub-study (1,217/1,333; 91.2%) had useable sleep data from at least 4 nights (nights did not have to be consecutive). Some participants provided data for more than 7 nights; all analyzable data were used in these cases.

Physical activity variables (covariates) were based on data from a separate waist-worn accelerometer (ActiGraph wGT2X-BT). Participants were instructed to wear the ActiGraph during all waking hours except for showering or swimming for 8 days; data was collected at 40 Hz each second with 60-s epochs and analyzed with daily diaries in hand. Participants had to wear the accelerometer for a minimum of 10 h over at least 4 days for data to be considered analyzable.

### Variables

#### Multidimensional sleep measures

Sleep variables derived from actigraphy in the present study included multiple standard dimensions of sleep health (duration, maintenance, regularity, and timing), physical activity, and sedentary time, which were averaged within-participant across the number of days of analyzable data (4–13 for sleep variables and 4–11 for physical activity). Total sleep time (*TST*), a measure of nocturnal sleep duration, was calculated as the total number of epochs scored as sleep within the time in bed period (i.e., the difference between the time at which the participant got into bed with the intention to go to sleep and the time at which they awoke in the morning, minus periods of wakefulness). Sleep maintenance was assessed through sleep efficiency (*SE*), time awake after sleep onset (*WASO*), and the Sleep Fragmentation Index (*SFI*). *SE* was calculated as *TST*/time in bed × 100. *WASO* was calculated as the total number of epochs scored as “awake” from sleep onset to time waking up in the morning. The *SFI*, an indicator of restlessness during the sleep period expressed as a percentage with higher scores indicating greater restlessness, is a composite score of two percentages; the movement index (number of scored epochs with one or more activity counts divided by total time in bed × 100) and the fragmentation index (percentage of 1-min periods of sleep vs. all periods of sleep in the period). Epochs were scored as movement if the number of activity counts recorded were greater than, or equal to, the epoch length in 15-s intervals [45]. Sleep timing was assessed using the midpoint of sleep, which was dichotomized into healthy and unhealthy timing. To calculate sleep midpoint, sleep onset and wake-up times were first converted to decimals and centered around midnight (negative values indicate times before midnight). Sleep midpoint was then calculated as: sleep onset time + ((wake-up time-sleep onset time)/2). For each participant, we calculated the average sleep midpoint across all nights with actigraphy. Consistent with previous SWAN analyses, the sleep midpoint variable was dichotomized as healthy (between 2:00 and 4:00 AM) or early/late timing (outside of 2:00–4:00 AM) [25]. Sleep regularity was determined by the *SD* of the midpoint from sleep onset to wake-up, in minutes, with greater values indicating greater irregularity in sleep timing.

#### Covariates

In addition to age, race/ethnicity, and study site, we considered covariates from a subset of sociodemographic, clinical, psychosocial, and behavioral factors collected from the SWAN parent and actigraphy sub-study with established theoretical relationships with sleep health for inclusion in multivariable models (displayed in Table 1). Covariates that had statistically significant relationships with at least one sleep variable (*p* < 0.05) were selected. To confirm the appropriateness of these variables (e.g., sufficient representation across groups) for inclusion in multivariable models, we also explored differences between BCS and controls on these factors. All covariates were collected or updated at visit 15 (either via daily sleep diaries or visit 15 parent interview), except for timing of anxiolytic use, which was derived from annual study visit data.

**Table 1:** Participant characteristics by BCS-control status (*N* = 1,110)

Characteristic	% (*N*) or median (*IQR*)		*p*-value
	BCS (*N* = 68)	Controls (*N* = 1,042)	
Sociodemographic and clinical characteristics			
*Age*	*65.5 (62.9, 68.0)*	*65.2 (63.3, 67.5)*	*0.58*
*Years since diagnosis*	*7.5 (3.9, 13.5)*	*7.8 (3.8, 14.2)*	*0.66*
*Race/ethnicity*			*0.49*
*White*	*45.6 (31)*	*45.1 (470)*	
*Black*	*25.0 (17)*	*25.7 (268)*	
*Hispanic*	*2.9 (2)*	*4.5 (47)*	
*Chinese*	*8.8 (6)*	*13.2 (138)*	
*Japanese*	*17.7 (12)*	*11.4 (119)*	
*Marital status*			*0.94*
*Currently married/living as married*	*58.8 (40)*	*60.6 (631)*	
*Never married*	*11.8 (8)*	*10.6 (110)*	
*Previously married*	*29.4 (20)*	*28.8 (300)*	
Educational level			0.30
Less than high school	4.4 (3)	4.7 (49)	
High school	10.3 (7)	15.3 (158)	
Some college	25.0 (17)	31.5 (326)	
College	23.5 (16)	22.7 (235)	
Post-graduate study	36.8 (25)	25.7 (266)	
*Difficulty paying for basic necessities*			*0.93*
*Not at all hard*	*81.5 (53)*	*80.4 (820)*	
*Somewhat hard*	*15.4 (10)*	*17.0 (173)*	
*Very hard*	*3.1 (2)*	*2.7 (27)*	
*Missing*	*3*	*22*	
Menopausal status			0.66
Natural postmenopause	94.1 (64)	92.4 (963)	
Bilateral oophorectomy	2.9 (2)	5.5 (57)	
Hysterectomy	2.9 (2)	2.1 (22)	
*Exogenous hormone use (yes/no)*	*30.9 (21)*	*4.2 (44)*	** < *0.0001***
*Type of exogenous hormone, users*			** < *0.0001***
*Systemic estrogen/progestin*	*0.0 (0)*	*63.6 (28)*	
*SERM*	*28.6 (6)*	*20.5 (9)*	
*Antineoplastic*	*66.7 (14)*	*0.0 (0)*	
*Unknown*	*4.8 (1)*	*15.9 (7)*	
*% of days with VMS*			*0.77*
*0*	*68.2 (45)*	*68.9 (703)*	
*1st non-zero tertile (*< *15%)*	*15.2 (10)*	*12.3 (125)*	
*2nd non-zero tertile (15–0%)*	*9.1 (6)*	*8.0 (82)*	
*3rd non-zero tertile (*> *40%)*	*7.6 (5)*	*10.8 (110)*	
*Missing*	*2*	*22*	
*Number of self-reported chronic comorbidities*			*0.65*
*0*	*11.8 (8)*	*9.1 (95)*	
*1*	*19.1 (13)*	*16.9 (176)*	
*2* +	*69.1 (47)*	*74.0 (771)*	
*Obstructive sleep apnea risk score*			*0.68*
*Low*	*31.9 (15)*	*38.3 (288)*	
*Intermediate*	*44.7 (21)*	*41.0 (309)*	
*High*	*23.4 (11)*	*20.7 (156)*	
*Missing*	*21*	*289*	
*Treated for sleep apnea (yes/no)*	*6.0 (4)*	*6.2 (64)*	*1.0000*
Behavioral and psychological factors			
*Timing of anxiolytics initiation*			***0.03***
*Never use*	*66.2 (45)*	*76.8 (800)*	
*First use post-diagnosis*	*13.2 (9)*	*5.8 (60)*	
*First use pre-diagnosis*	*20.6 (14)*	*17.5 (182)*	
*Reported use of prescription or OTC sleep medications in sleep diaries*			*0.18*
*None*	*74.2 (49)*	*80.6 (820)*	
*1st tertile (*≤ *25% of nights)*	*4.6 (3)*	*6.3 (64)*	
*2nd tertile (25.1–87.5% of nights)*	*15.2 (10)*	*7.5 (76)*	
*3rd tertile (*> *87.5% of nights)*	*6.1 (4)*	*5.7 (58)*	
*Any naps 30* + *min* (*yes/no*)	*53.0 (35)*	*50.5 (506)*	*0.68*
*Missing*	*2*	*38*	
Smoking			0.60
Never	58.5 (38)	64.6 (667)	
Past	35.4 (23)	30.2 (312)	
Current	6.2 (4)	5.1 (53)	
Missing	3	10	
*Alcohol, # servings/week*			*0.05*
*0*	*41.8 (28)*	*51.6 (533)*	
< *1/week*	*32.8 (22)*	*22.8 (235)*	
*1–7/week*	*11.9 (8)*	*17.9 (185)*	
> *7/week*	*13.4 (9)*	*7.7 (80)*	
*Missing*	*1*	*9*	
*Mean daily MVPA minutes (NHANES)*	*10.0 (4.7, 22.3)*	*10.7 (4.5, 21.4)*	*0.83*
*Median daily sedentary time (minutes)*	*590 (530, 687)*	*608 (538, 675)*	*0.61*
*Generalized anxiety disorder score*	*1 (0, 4)*	*1 (0, 4)*	*0.32*
*CES-D depression*	*7.4 (5)*	*10.9 (113)*	*0.54*
*Missing*	*0*	*1*	

Italic emphasis indicates covariates selected for inclusion in adjusted models

Demographic covariates considered for model inclusion were age, self-reported race/ethnicity (White, Black, Chinese, Hispanic, and Japanese), difficulty paying for basic necessities (not at all, somewhat, or very hard), education level (less than high school, high school, some college, college, and post-graduate study), and marital status (currently/living as, never, or previously married). Clinical characteristics included years since diagnosis (or pseudo-diagnosis), menopausal status (natural post-menopausal, bilateral oophorectomy, and hysterectomy), the proportion of nights with any vasomotor symptoms from sleep diaries (VMS; hot flashes or night sweats), timing of anxiolytic-based sleep medication (e.g., barbiturate or benzodiazepine use; none, pre-diagnosis, and post-diagnosis), the proportion of nights using prescription or over-the-counter sleep medications from sleep diaries (none, 1st, 2nd, and 3rd tertile), Obstructive Sleep Apnea Risk Score (low, intermediate, or high [46]), whether participants reported being treated for sleep apnea (yes/no), and number of self-reported chronic medical comorbidities (trichotomized into 0, 1, or 2 +).

Behavioral and psychological covariates considered for model inclusion were alcohol consumption (abstain, < 1 serving/week, 1–7 servings/week, and > 7 servings/week), smoking (never, past, and current), depressive symptoms, anxiety symptoms, naps exceeding 30 min (yes/no), minutes of moderate-vigorous physical activity, and sedentary time. Depressive symptoms over the last week were measured using the Center for Epidemiological Studies Depression Scale (CES-D), with higher scores indicating more depressive symptoms [47]. Anxiety symptoms over the last 2 weeks were measured using the Generalized Anxiety Disorder-7 Scale (GAD-7) [48], with higher scores indicating greater anxiety. Naps were characterized as yes/no (whether they reported any naps in sleep diaries throughout the week that lasted longer than 30 min; no naps > 30 min vs. 1 + naps > 30 min). Light, moderate, and vigorous intensity physical activity were obtained from waist-worn actigraphy and were distinguished using established cut points from NHANES [49]. Analyses used the mean daily minutes of MVPA averaged across days of wear for at least 10 h. Mean daily sedentary minutes included all minutes with activity counts < 100 across days of wear.

### Statistical analyses

Characteristics were compared for BCS and controls using chi-square or Fisher’s exact tests for categorical variables and Wilcoxon rank sum tests for continuous variables. To address our first objective comparing BCS and controls on actigraphy-derived sleep health, we first regressed sleep duration (*TST*), sleep maintenance (*SE*, *WASO*, and *SFI*), sleep regularity, and sleep timing (healthy sleep midpoint) onto breast cancer status in unadjusted models, using binomial logistic regression for healthy sleep midpoint and linear regression for all other sleep outcomes. Corresponding covariate-adjusted BCS-control differences were estimated, adding covariates that were significantly associated with one or more sleep outcomes in bivariate analyses, as well as age, race/ethnicity, and site. In post hoc analyses, we also identified significant predictors of sleep variables in adjusted models. To address our second objective of whether BCS-control differences were modified by years since diagnosis, we tested the interaction between breast cancer status and years since diagnosis/pseudo-diagnosis in these models in sleep outcomes, before and after covariate adjustment, using 5 years as a cut-point to distinguish early vs. late breast cancer survivorship. Several outcomes — sleep maintenance (SE, *WASO*, and *SFI*), and regularity — were left- or right-skewed, but results after transforming to obtain normally distributed residuals were consistent; thus, we present results for untransformed outcomes to maximize interpretability. We also ran several sensitivity analyses: (1) reducing the number of model covariates using backward elimination, (2) excluding BCS 10 or more years post-diagnosis, (3) excluding BCS experiencing a recurrence or second cancer and controls with a cancer diagnosis other than breast, and (4) without New Jersey participants (because this site did not collect data at visit 8). The results were consistent across main and sensitivity analyses; thus, we present models inclusive of the greatest number of participants in the “Results” section.

## Results

### Sample characteristics

The median time since diagnosis for BCS was 7.5 years; 64% of BCS were more than 5 years post-diagnosis. BCS and controls did not differ on key sociodemographic or sleep, behavioral, or psychosocial characteristics, (*p*’s > 0.05, see Table 1). BCS had significantly greater use of exogenous hormones (*p* < 0.0001) due to their use of selective estrogen receptor modulators (SERMs) and antineoplastic exogenous hormones (frequently prescribed as part of breast cancer treatment [50]). Although BCS and controls reported similar rates of using over the counter or prescription sleep medications in the daily sleep diaries (collected concurrently with actigraphy) and current use of anxiolytic sleep medications at visit 15 (which may include benzodiazepines prescribed for an indication other than sleep), they were more than twice as likely to have initiated anxiolytic use for the first time post-diagnosis compared to controls post-pseudo-diagnosis (*p* = 0.03; Table 1). Among those who reported ever using anxiolytic medications (33.8% of BCS and 23.3% of controls), the number of annual study visits using anxiolytics was similar across both groups (median of 2 visits, range of 1–15, most using between 1 and 5 visits).

### Actigraphy measures

A significant proportion of the overall sample (*N* = 1,110) reported sub-optimal sleep health across various dimensions: mean total sleep time (*TST*) was 6.48 h (*SD* = 0.04), mean sleep efficiency (*SE*) was 81.95% (*SD* = 0.24%), mean wake after sleep onset (*WASO*) was 51.66 min (*SD* = 0.83), mean Sleep Fragmentation Index (*SFI*) was 32.06% (*SD* = 0.54%), mean sleep regularity was 46.96 min (*SD* = 1.38), and 32% had unhealthy sleep timing (midpoint outside 2–4 AM). In unadjusted models, BCS and controls did not differ on any of the actigraphy-derived sleep metrics. The results were consistent following covariate adjustment (all *p*’s > 0.1; Table 2).

**Table 2:** Covariate-adjusted^a^. Differences in sleep characteristics between breast cancer survivors (BCS; *N* = 63) and controls (*N* = 970)

Sleep outcome	*Mean* (*SE*)		*p*-value
	BCS	Control	
Total sleep time (*TST*; hours)	6.44 (0.12)	6.49 (0.03)	0.68
Sleep efficiency (*SE*; percent of time in bed asleep)	80.91 (0.82)	82.21 (0.21)	0.12
Wake after sleep onset (*WASO*; minutes)	53.02 (2.82)	50.83 (0.71)	0.45
Sleep Fragmentation Index (*SFI*; percent of sleep period with events)	32.89 (1.84)	32.01 (0.46)	0.64
Sleep regularity (*SD* midpoint; minutes)	39.00 (4.20)	46.8 (1.20)	0.09
Dichotomized healthy midpoint	**% (*SE*)**		
Not between 2:00 and 4:00AM	Reference		
Between 2:00 and 4:00AM	60.40 (6.65)	71.19 (1.53)	0.09

^a^Adjusted for age, race/ethnicity, site, marital status, difficulty paying for basic necessities, categorized # comorbidities, VMS frequency from actigraphy diaries, percentage of nights using OTC/Rx sleep medication from actigraphy diaries, timing of Rx anxiolytic use (from annual visit data), MVPA, sedentary time, any naps 30 + min, GAD, CES-D depression, and treatment for sleep apnea

Note: Results consistent with unadjusted models and sensitivity analyses (excluding BCS and controls 10 + years post-diagnosis/pseudo-diagnosis, excluding BCS experiencing a recurrence or second cancer, excluding controls with a cancer diagnosis other than breast, and excluding New Jersey participants)

Covariates that were associated with one or more sleep outcomes in bivariate (unadjusted) analyses and included in adjusted models were race/ethnicity, marital status, and difficulty paying for basic necessities, VMS, comorbidities, sleep medication use, timing of anxiolytic medication use, naps > 30 min, alcohol consumption, sedentary time, MVPA time, anxiety, depression, treatment for sleep apnea, and obstructive sleep apnea risk. In adjusted models, several covariates remained significantly associated (*p* < 0.05) with multiple sleep dimensions: Black race (compared with non-Hispanic white), napping > 30 min, greater VMS frequency, greater depressive symptoms, reported sleep apnea treatment, and sedentary time were each associated with greater sleep disturbance, while greater MVPA was associated with lower sleep disturbance (Table 3). Both pre- and post-diagnosis anxiolytic uses were associated with longer *TST* compared to never use (pre-diagnosis *b* = 14.88, *SE* = 4.83, post-diagnosis *b* = 5.64, and *SE* = 7.38; *p* = 0.009).

**Table 3:** Covariates from multivariable models statistically significantly associated with sleep characteristics (*N* = 63 BCS, 970 controls)

**Total sleep time (*TST*); unit = minutes**		
**Covariate**^**a**^	**Coefficient (*SE*)**	**Overall *p*-value**
Race/ethnicity		< 0.0001
White	Reference	
Black	− 20.14 (5.07)****	
Chinese	− 30.15 (8.58)***	
Hispanic	10.48 (16.92)	
Japanese	− 33.39 (8.28)****	
Difficulty paying for basics		0.04
Not at all	Reference	
Somewhat	− 5.51 (5.76)	
Very	− 29.59 (12.17)*	
Actigraphy nights with sleep medication use		0.01
None	Reference	
1st tertile	− 1.65 (7.15)	
2nd tertile	13.46 (6.48)*	
3rd tertile	20.51 (7.56)**	
Timing of anxiolytic use		0.009
Never	Reference	
Post-diagnosis	5.64 (7.38)	
Pre-diagnosis	14.88 (4.83)**	
Any naps 30 + min	− 7.33 (3.51)*	0.04
Sedentary time	− 0.11 (0.02)****	< 0.0001
MVPA	− 0.26 (0.12)*	0.03
**Sleep efficiency (*SE)*; unit = percent of time in bed asleep**		
Race/ethnicity		< 0.0001
White	Reference	
Black	− 4.45 (0.60)****	
Chinese	− 1.18 (1.02)	
Hispanic	− 1.35 (2.01)	
Japanese	− 1.34 (0.99)	
VMS from actigraphy diaries		0.009
None	Reference	
1st tertile	− 1.63 (0.62)**	
2nd tertile	− 1.49 (0.75)*	
3rd tertile	− 1.40 (0.70)*	
# comorbidities		0.02
0	Reference	
1	− 2.05 (0.81)*	
2 +	− 1.86 (0.72)**	
CES-D depression	− 2.76 (0.81)***	0.0007
**Wake after sleep onset (*WASO*); unit = minutes**		
Race/ethnicity		0.0007
White	Reference	
Black	8.50 (2.07)****	
Chinese	− 4.89 (3.51)	
Hispanic	− 2.27 (6.92)	
Japanese	− 0.53 (3.38)	
VMS from actigraphy diaries		0.0026
None	Reference	
1st tertile	2.90 (2.14)	
2nd tertile	6.15 (2.59)*	
3rd tertile	7.58 (2.41)**	
Sedentary time	− 0.025 (0.006)****	< 0.0001
MVPA	− 0.104 (0.047)*	0.03
CES-D depression	7.03 (2.79)*	0.01
**Sleep Fragmentation Index (*SFI*); units = percent of sleep period with events**		
Marital status		0.049
Currently married/living as married	Reference	
Never married	1.58 (1.53)	
Previously married	2.66 (1.10)*	
CES-D depression	4.31 (1.82)*	0.02
Treatment for sleep apnea	6.01 (1.96)**	0.002
**Regularity; units = minutes**		
Any naps 30 + min	0.11 (0.04)**	0.004
Sedentary time	0.0006 (0.0002)***	0.0003
MVPA	− 0.0034 (0.0012)**	0.005
Treatment for sleep apnea	0.15 (0.08)*	0.047
**Healthy midpoint of sleep (between 2:00 and 4:00AM)**		
**Covariate**^**a**^	**Odds ratio (95% *CI*)**	**Overall *p*-value**
Marital status		0.03
Currently married/living as married	Reference	
Never married	0.69 (0.44, 1.08)	
Previously married	1.33 (0.94, 1.87)	
Sedentary time	0.999 (0.997, 1.000)*	0.02
MVPA	1.02 (1.01, 1.03)**	0.002
CES-D depression	0.39 (0.23, 0.66)***	0.0005

* *p* < 0.05; ** *p* < 0.01; *** *p* < 0.001; **** *p* < 0.0001

^a^Only covariates significantly associated with each sleep outcome are presented in this table; however, all adjusted models included the same covariates (age, race/ethnicity, and site, marital status, difficulty paying for basic necessities, categorized % diary days with night sweats, categorized # comorbidities, categorized % diary days with OTC or Rx sleep meds, timing of Rx anxiolytic use (from annual visit data), any naps 30 + min, alcohol consumption, sedentary time, moderate/vigorous physical activity, GAD anxiety, CES-D depression, and treatment for sleep apnea)

To address the second objective, we next examined how time since diagnosis impacted BCS-control differences in actigraphy-derived sleep by testing interactions between cancer status and years since diagnosis. We found that the number of years since diagnosis significantly modified the BCS-control difference in mean *TST* (*p* = 0.02; Table 4). Specifically, BCS within 5 years of diagnosis had lower *TST* compared to controls (6.13 h vs. 6.57 h; *p* = 0.03), but BCS more than 5 years post-diagnosis did not differ significantly from controls in their *TST* (6.59 h vs. 6.45 h; *p* = 0.32). Except for *TST*, there was no statistically significant effect modification of BCS-control differences by time since diagnosis.

**Table 4:** Covariate-adjusted^a^ BCS-control differences in actigraphy outcomes, by years since diagnosis/pseudo-diagnosis — split at ≤ 5 years vs. > 5 years; 63 BCS (21 early; 42 late) and 970 controls (306 early; 664 late)

Sleep outcome	*Mean* (*SE*)		*p*-value, BCS vs. control	*p*-value, BCS/control × years since diagnosis
	BCS (*N* = 63; 21 ≤ 5 years, 42 > 5 years)	Controls (*N* = 970; 306 ≤ 5 years, 664 > 5 years)		
Nightly total sleep time (*TST*; hours)				**0.02**
≤ 5 years post-diagnosis	6.13 (0.20)	6.57 (0.05)	**0.03**	
> 5 years post-diagnosis	6.59 (0.14)	6.45 (0.04)	0.32	
Mean sleep efficiency: (*SE*; percent of time in bed asleep)				0.14
≤ 5 years post-diagnosis	79.42 (1.42)	82.49 (0.37)	**0.04**	
> 5 years post-diagnosis	81.66 (1.02)	82.08 (0.25)	0.69	
Wake after sleep onset (*WASO*; minutes)				0.97
≤ 5 years post-diagnosis	52.76 (4.88)	50.36 (1.29)	0.63	
> 5 years post-diagnosis	53.18 (3.50)	51.05 (0.86)	0.55	
Sleep fragmentation (*SFI*; percent of sleep period with events)				0.70
≤ 5 years post-diagnosis	33.60 (3.17)	31.66 (0.84)	0.56	
> 5 years post-diagnosis	32.54 (2.28)	32.16 (0.56)	0.87	
Regularity (minutes)				0.46
≤ 5 years post-diagnosis	43.80 (7.80)	46.80 (1.80)	0.70	
> 5 years post-diagnosis	36.60 (5.4)	46.20 (1.20)	0.07	
Healthy midpoint (percentage with midpoint between 2:00 and 4:00AM)	**% (*SE*)**			0.40
≤ 5 years post-diagnosis	68.64 (10.87)	71.22 (2.72)	0.82	
> 5 years post-diagnosis	56.08 (8.39)	71.17 (1.85)	0.06	

^a^Adjust for all covariates significantly associated (*p* < 0.05) with 1 + sleep outcome in bivariate analyses, forcing in age, race/ethnicity, and site; *N* = 1033: marital status, difficulty paying for basic necessities, categorized % diary days with night sweats, categorized # comorbidities, categorized % diary days with OTC or Rx sleep meds, timing of Rx anxiolytic use (from annual visit data), any naps 30 + min, alcohol consumption, sedentary time, moderate/vigorous physical activity, GAD anxiety, CES-D depression, and treatment for sleep apnea

Note: Covariate-adjusted patterns are generally consistent with corresponding unadjusted patterns; results consistent with/without BCS and controls 10 + years post diagnosis/pseudo-diagnosis, BCS experiencing a recurrence or second cancer, controls with a cancer diagnosis other than breast, and New Jersey participants

## Discussion

In this cohort of 68 breast cancer survivors (BCS) and 1,042 non-cancer controls, we found few differences in actigraphy-estimated dimensions of sleep health or psychosocial and behavioral risk factors for sleep disturbance. There were no significant differences between BCS (most of whom were at least 5 years post-diagnosis) and controls in adjusted or unadjusted models predicting sleep duration (total sleep time), maintenance (sleep efficiency, wake after sleep onset, and sleep fragmentation), regularity, or timing.

In covariate-adjusted analyses by time since diagnosis, we found that BCS within 5 years of diagnosis exhibited shorter total sleep time compared to controls, but this difference was no longer significant among longer-term survivors. Our finding that BCS who were likely predominantly post-treatment and within 5 years of diagnosis showed shorter sleep duration is in contrast to previous longitudinal studies using actigraphy, one showing no BCS-control differences in sleep duration during or after treatment [9] and another showing reductions in BCS’ sleep duration after chemotherapy initiation that returned to baseline levels no different than controls within 4 cycles of chemotherapy [27]. While the present study includes sensitivity analyses excluding BCS who experienced a recurrence, we lack precise information regarding the timing of assessments with respect to completion of other treatments such as chemotherapy or radiation. Thus, it is possible that the lower sleep duration among BCS within 5 years of diagnosis was due to some BCS in this study still receiving or having recently completed active treatment. Overall, our findings that BCS experience few long-term differences in actigraphy-measured sleep health domains compared to controls suggest that any breast cancer treatment-related disruptions in actigraphy-estimated sleep may eventually improve farther out from treatment.

We also did not find any BCS-control differences in daytime napping or sedentary activity. Increased napping and sedentary activity have been identified as key perpetuating factors for sleep disturbance and a link between cancer-related fatigue and insomnia. Although we did not directly measure fatigue, the fact that we did not see BCS-control differences in napping and inactivity is consistent with research showing that fatigue and daytime napping typically return to baseline levels within 1–2 years of treatment completion among BCS [9, 51]. However, naps, moderate to viorous physical activity, and sedentary activity were significant predictors of multiple dimensions of sleep disturbance in adjusted models, suggesting that these factors are important considerations in sleep treatment for both BCS and similarly-aged women without breast cancer. Depressive symptoms, frequency of vasomotor symptoms, and treatment for sleep apnea were also related to multiple facets of sleep health, indicating that identifying and addressing these issues may be important for improving sleep health regardless of cancer history. Surprisingly, exogenous hormone use was not associated with any aspect of sleep disturbance measured in this study, in contrast to previous research linking endocrine therapy to greater sleep disturbances among BCS [10].

Although BCS and controls reported similar levels of over-the-counter and prescription sleep medication use in their daily diaries and anxiolytic medications at visit 15, BCS were more than twice as likely to have initiated anxiolytic medication use post-diagnosis compared to controls (13.2% vs. 5.8%). Use of anxiolytics among BCS years post-diagnosis warrants greater clinical focus, as many these medications are not indicated for long-term use and have harms that outweigh benefits in many cases. BCS and controls who reported use of anxiolytic medications took them for a median of 2 annual visits, with some reporting use across as many as 15 annual visits, suggesting that long-term anxiolytic use is common in this population. Anxiolytic use initiated either before or after diagnosis was associated with only modestly longer sleep duration (approximately 6 more min for post-diagnosis initiation and 15 min for pre-diagnosis initiation) and was not associated with sleep maintenance, timing, or regularity. Given the modest potential benefit of post-diagnosis anxiolytic use for sleep duration and similar rates of self-reported use at the time of actigraphy data collection, it is unlikely that these medications masked any case–control differences.

Despite a lack of overall difference between BCS and controls on actigraphy-derived measures of sleep, sleep health was sub-optimal for both groups, suggesting a need to address these issues within the broader population of mid-life and older adult women. Indeed, across our sample, participants slept approximately 6.5 h, which is less than the 7–8 h recommended for older adults [52]; their sleep was relatively inefficient at 82% (below the ≥ 85% threshold for good sleep consolidation); they spent nearly an hour awake during the night, suggesting poor sleep quality; their sleep was significantly restless per an average fragmentation index score of 32%; and more than 30% had an unhealthy sleep midpoint [53, 54]. Although our sample sleep parameters on average are comparable to meta-analytic findings for similarly-aged adults without clinical insomnia [55], they are also consistent with population-based research demonstrating a high prevalence of short sleep duration and insomnia symptoms among adults older than 65 (approximately one-third achieving < 7 h of sleep, 20% regularly using sleep aids) [56]. Taken together, these findings suggest that enhanced sleep health screening, education, and intervention for mid-life and older adult women are warranted.

The results of this study should be considered within the context of its limitations. Although we captured multidimensional sleep health among a cohort of BCS with varying time since diagnosis, we lack information on sleep health closer to diagnosis and treatment, when disruptions in sleep may be more pronounced. Actual (as opposed to actigraphy-assessed) BCS-control differences in sleep health may be greater than what we observed in the present study. Compared with PSG, actigraphy tends to overestimate sleep duration and maintenance (overestimating total sleep time and efficiency and underestimating wake after sleep onset); these biases appear larger among individuals with chronic illnesses compared to healthy controls [57]. Additionally, because the present study involved a research cohort with age-related inclusion criteria, there were no participants younger than 60 in the sample. Thus, we lack information about the sleep health of younger cancer survivors, who experience greater actigraphy-derived and self-reported sleep difficulties [4, 58, 59]. Previous work suggests that cancer-related disruptions to mood and sleep may be especially pronounced for BCS younger than 45 at diagnosis or pre-menopausal during treatment [59, 60]. It is possible that differences in actigraphy-measured sleep would be greater among a cohort of younger women and that the effect of cancer diagnosis and treatment on sleep may not be as pronounced in this population of women mostly in their 60’s. More work is needed to understand the specific mechanisms that may make younger women more vulnerable to the effects of diagnosis and treatment. Data from the present study were also collected prior to the COVID-19 pandemic and the subsequent societal shift towards working from home; it is unclear how changes in sleep associated with working from home (e.g., later bedtimes, longer naptime and total sleep time, and increased regularity [61–63]) might impact this particular population or change any BCS-control differences. Another limitation of our study is that we focused solely on sleep health and did not assess circadian rest-activity rhythms. Future research should compare BCS and controls on circadian rest-activity patterns to better understand long-term consequences of diagnosis and treatment and gain a more comprehensive picture of overall circadian health among BCS.

Lastly, our sample of 68 BCS is relatively small. While this is a reasonable number for actigraphy studies, and the study is powered to detect a small to medium effect size in terms of BCS-control differences (detectable mean difference = 0.355), analyses are underpowered to detect within-BCS differences by time since diagnosis. Analyses were adjusted for a relatively large number of covariates given the sample size for BCS, but these BCS-control differences are highly consistent with both differences unadjusted for covariates and with results from sensitivity analyses that reduced the number of model covariates using backward elimination (data not shown); thus, BCS-control differences presented here should not be affected by possible model overfitting.

This study has numerous strengths, including being the largest to date in the published literature to compare actigraphy-derived sleep health and behavioral risk factors between BCS and controls of similar age, race/ethnicity, SES, comorbidities, and menopausal status. This study had an equivalent number of BCS and over 10 times as many controls as the largest comparison of BCS and controls on actigraphy-derived sleep to date [9]. This population also has greater racial/ethnic diversity than previous samples, additional actigraphy sleep parameters included (timing and regularity, maintenance), a greater number of covariates considered, and rigorous actigraphy data collection methods. Additionally, although analyses were cross-sectional and assessed only actigraphy-derived measures of sleep health, the lack of difference in actigraphy-derived measures is consistent with longitudinal findings using a single-item subjectively-rated measure of sleep maintenance in the same population [20]. This previous SWAN study by Goyal and colleagues found no differences in sleep disturbance between BCS and controls pre- or post-diagnosis and found that sleep problems worsened within the 2 years prior to diagnosis rather than after, providing additional support for our findings that BCS in this diverse, well-matched population exhibit similar sleep characteristics.

## Conclusions

BCS largely demonstrated similar rates of poor actigraphy-derived sleep health compared with similar-aged women without breast cancer, although BCS within the first 5 years of diagnosis experienced shorter total sleep time. BCS and controls overall experienced poor sleep health, suggesting enhanced screening and treatment is warranted for this population regardless of cancer history.

## Data Availability

The datasets generated during and/or analyzed during the current study are available from the corresponding author on reasonable request.
